# Critical care provision after colorectal cancer surgery

**DOI:** 10.1186/s12871-016-0243-9

**Published:** 2016-10-12

**Authors:** C. D. Dale, P. McLoone, B. Sloan, J. Kinsella, D. Morrison, K. Puxty, T. Quasim

**Affiliations:** 1 Undergraduate Medical School, School of Medicine, University of Glasgow, Glasgow, UK; 2 West of Scotland Cancer Surveillance Unit, Public Health Research Group, Institute of Health and Wellbeing, University of Glasgow, Glasgow, UK; 3 Anaesthesia, Critical Care and Pain Medicine, School of Medicine, Glasgow Royal Infirmary, Glasgow, Scotland, UK

**Keywords:** Colorectal cancer, Surgery, Organ support, ICU

## Abstract

**Background:**

Colorectal cancer (CRC) is the 2nd largest cause of cancer related mortality in the UK with 40 000 new patients being diagnosed each year. Complications of CRC surgery can occur in the perioperative period that leads to the requirement of organ support. The aim of this study was to identify pre-operative risk factors that increased the likelihood of this occurring.

**Methods:**

This is a retrospective observational study of all 6441 patients who underwent colorectal cancer surgery within the West of Scotland Region between 2005 and 2011. Logistic regression was employed to determine factors associated with receiving postoperative organ support.

**Results:**

A total of 610 (9 %) patients received organ support. Multivariate analysis identified age ≥65, male gender, emergency surgery, social deprivation, heart failure and type II diabetes as being independently associated with organ support postoperatively. After adjusting for demographic and clinical factors, patients with metastatic disease appeared less likely to receive organ support (*p* = 0.012).

**Conclusions:**

Nearly one in ten patients undergoing CRC surgery receive organ support in the post operative period. We identified several risk factors which increase the likelihood of receiving organ support post operatively. This is relevant when consenting patients about the risks of CRC surgery.

## Background

In the UK 40,000 people are diagnosed with colorectal cancer (CRC) annually and it is the second most common cause of cancer related death [1]. Mortality attributable to CRC has decreased by approximately 40 % since the 1970s with the introduction of bowel screening, better surgical techniques and adjuvant chemotherapy [1].

CRC surgery is associated with many early post-operative complications including wound infections, persistent ileus, bleeding, anastomotic leak, pneumonia, urinary tract infections, thromboembolism and cardiac complications [2]. These complications along with pre-existing co-morbidities are the commonest reasons for requirement of post-operative organ support. One small, single centre study, has reported that CRC made up 3 % of admissions to intensive care (ICU). In that study, metabolic, haemodynamic and cardiovascular complications were the leading causes of admission [3].

Acute complications following colorectal cancer surgery are associated with a detrimental effect on short and long term survival. The most notable of these complications is an anastomotic leak, which occurs in 3–14 % [4–8] of patients. It carries a short term mortality of 7–18 % [8–10] and is associated with poor long term cancer outcomes in terms of both survival [5, 11, 12] and recurrence [13, 14]. This complication generally occurs 6 days post-operatively and has a wide spectrum of presentations. Whilst many small leaks remain subclinical, for some it presents with life-threatening intra-abdominal sepsis and multi-organ failure requiring ICU admission for organ support.

Many studies have tried to determine the risk factors associated with postoperative morbidity and mortality in this population. They include male sex [15–20], increasing age [16, 21–25], American Society of Anesthesiologists (ASA) Physical Status score [22, 24, 25], co-morbidities [16, 20, 21, 26–29], advanced tumour stage [15, 22, 24, 25, 29], neoadjuvant chemotherapy [15], emergency surgery [21, 22, 24, 25], proximity of surgery to the anal verge [15, 18, 19] and social deprivation [30]. However, there was a wide variety in the ways in which morbidity and mortality were described through the literature and many of the studies were from single centres with small sample sizes.

As our population becomes increasingly elderly, more patients with significant co-morbidities will be considered for surgical intervention. No study, to our knowledge, has reported the proportion of patients requiring organ support or the type of organ support required, following CRC surgery. Identifying patients who were likely to require organ support would allow us to optimise the patient journey and appropriately consent high-risk patients.

## Methods

### Study design

**Fig. 1:**
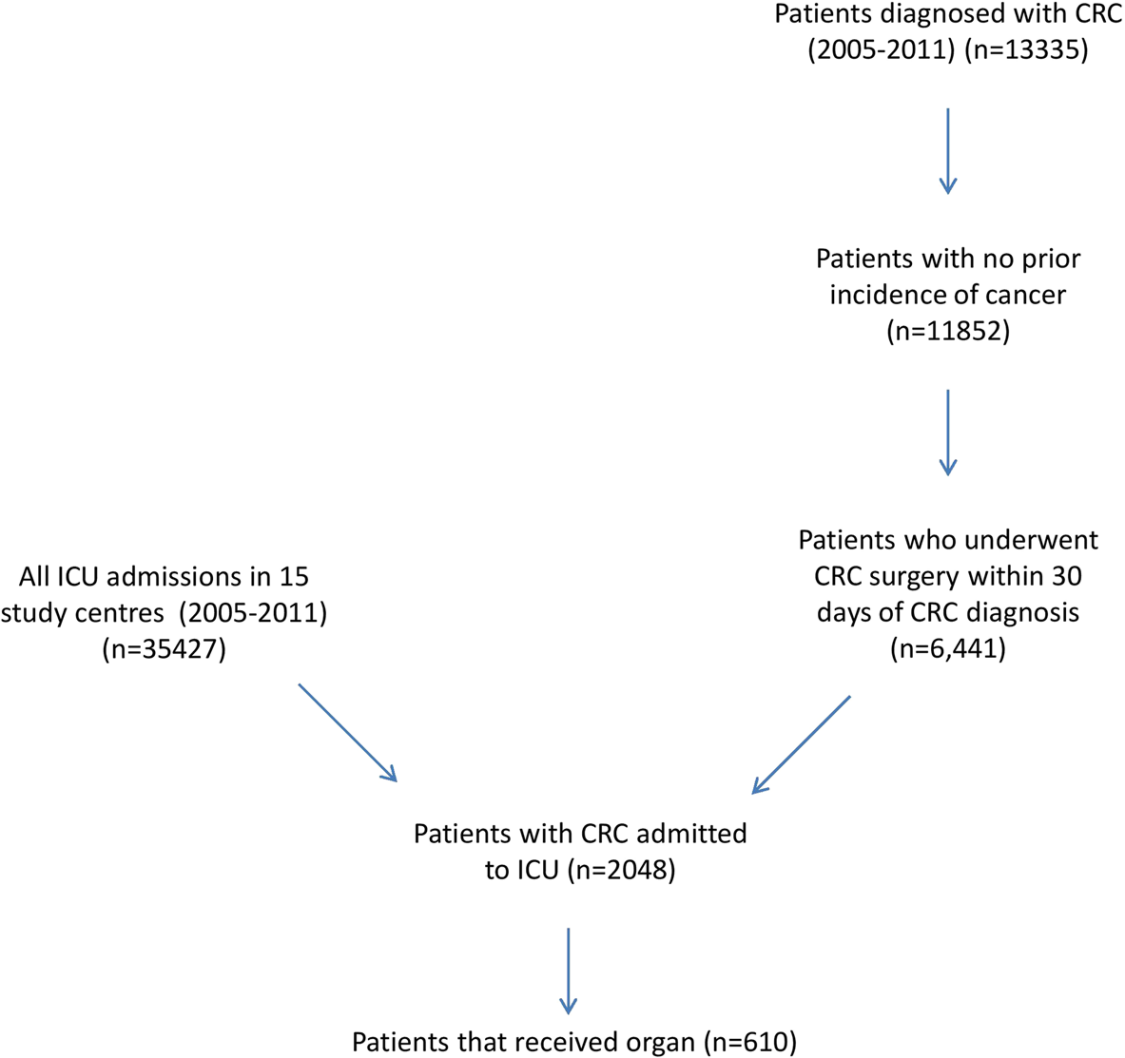
Consort diagram of cohort

This is a multi-centre, retrospective, observational study assessing which pre-operative factors predict receipt of organ support following CRC surgery. We identified patients resident in the West of Scotland region that had a diagnosis of a CRC on the Scottish Cancer Registry between 2005 and 2011 and determined whether they had undergone CRC surgery. We then established which of these patients were admitted to ICU for organ support in the post-operative period. (See Fig. 1)

### Data

The Scottish Cancer Registry collects information on all new cases of cancer including primary malignant neoplasms, carcinoma in-situ, neoplasms of uncertain behaviour and benign brain and spinal cord tumours. Cancer diagnoses are coded to the International Classification of Diseases 10th revision (ICD-10). The registry is linked by the Information Services Division (ISD) of NHS Scotland to Scottish Morbidity records (SMR01) which detail all hospital discharges, National Records of Scotland death records and to The Scottish Intensive Care Society Audit Group (SICSAG) WardWatcher (Critical Care Audit Ltd, Otley, Yorkshire) ICU audit database.

The SICSAG WardWatcher audit system is a national database used in Scotland to collect data on patient demography, admitting specialty and diagnosis, patient’s prior location, known co-morbidities and type of organ support for all ICU admissions. Organ support was defined as invasive mechanical ventilation via endotracheal tube or tracheostomy, use of inotropic or vasopressor medication or renal replacement therapy (RRT) of any modality. We used the Scottish Index of Multiple Deprivation (SIMD) to describe deprivation by geographical location ranking areas from most deprived (1st quintile) to least deprived (5th quintile). Previous research using this index has shown that a higher level of socioeconomic deprivation is associated with poorer survival following colorectal cancer surgery [31]. This is thought to be due to higher early postoperative mortality in patients from more deprived areas [32]. Co-morbidities were identified using the SMR 01 data where hospitalisations in the previous 5 years were identified and the diagnosis field was searched for any of the following: myocardial infarction (MI)/ coronary heart disease (CHD), congestive cardiac failure (CCF), chronic obstructive pulmonary disease (COPD)/ asthma, or type II diabetes mellitus (TIIDM).

### Population

The West of Scotland has a population of 2.4 million. It is predominantly urban with the majority of the population living within the city of Glasgow or in large towns. There were 15 general ICUs in the area during the study period. Some functioned as combined ICU/ High Dependency Units (HDU) for some or all of the time period.

### Inclusion criteria

We defined patients as having CRC if they were resident in the West of Scotland and had a diagnosis of CRC (ICD-10 C18.0-18.9, C19 and C20) in the Scottish Cancer Registry between 1st January 2005 and 31st December 2011. SMR-01 data relating to any hospitalisation within 3 months of cancer incidence identified patients who had received CRC surgery (OPCS codes H04-H11, H13, H15, H29, H33, X14).

Patients admitted to ICU during the same inpatient stay associated with their colorectal surgery were identified. The Ward Watcher database records of these patients were then used to ascertain whether one of mechanical ventilation, vasopressor therapy or renal replacement therapy was required during their stay in ICU. If the patient had more than one ICU admission at any point in their cancer journey we only used data from the index admission.

Following any surgical procedure it is routine practice to transfer the patient to a recovery area until the patient has recovered from anaesthesia. Once stable the patient is then transferred to the surgical ward or high dependency unit. It is not routine practice to admit post-operative patients to ICU unless there is ongoing organ dysfunction requiring the provision of organ support.

### Emergency and elective surgery patients

The type of the hospital admission was determined using SMR-01 codes for either elective or emergency hospital admissions. If an admission was associated with only 1 surgical procedure during the hospital stay then the patient’s surgery was determined to be the same as the type of hospital admission (either elective or emergency). If, however, a patient was admitted to hospital electively but underwent more than 1 surgical procedure then the patient was classified as an “Emergency Surgery” patient. These patients were deemed to have required emergency surgery following their initial elective surgery e.g., for the repair of an anastomotic leak.

### Statistical analysis

Continuous variables were summarised as medians with interquartile range. *X* ^2^ test of association was used to assess differences in baseline characteristics between those who received organ support and those who did not. Multiple logistic regression analysis was used to test associations between receipt of organ support and demographic and clinical variables and receipt of organ support. Only variables that would have been known prior to surgery were used for logistic regression analysis. All variables were included in both univariate and multivariate analysis. Results were considered to be statistically significant if the *p*-value was <0.05. Statistical analyses were performed using Stata (Stata Statistical Software: Release 12. College Station, TX).

## Results

### Patient demographics

A total of 6,441 (median age 70 (IQR 62–77), 53 % male) patients underwent surgery for CRC during the study period. Post-operative organ support was provided to 610 patients (9 %) with a median age 71 (IQR 64–68), of whom 61 % male. Patient demographics and the proportions who received organ support are reported in Table 1.

**Table 1:** Characteristics of patients undergoing colorectal cancer surgery categorised on their requirement for postoperative organ support

Baseline Characteristic		All patients (*n* = 6441)	Number receiving organ Support (*n* = 610)	Percent receiving organ support (95 % CI)	*p*-value*
Sex	Men	3444 (53)	371 (61)	10.8 (9.8–11.9)	<0.001
	Women	2997 (47)	239 (39)	8.0 (7.0–9.0)	
Age (yrs)	Median	70 (62–77)	71 (64–78)		
Age group (yrs)	≥65	4384 (68)	455 (75)	10.4 (9.5–11.3)	<0.001
	<65	2057 (32)	155 (25)	7.5 (6.4–8.7)	
SIMD quintile	1 -most deprived	1718 (27)	176 (29)	10.2 (8.9–11.8)	0.023
	2	1519 (24)	161 (26)	10.5 (7.2–10.0)	
	3	1240 (19)	119 (20)	9.6 (8.0–11.3)	
	4	973 (15)	85 (14)	8.7 (7.0–10.7)	
	5 - least deprived	991 (15)	69 (11)	7.0 (5.5–8.7)	
Surgery type	Elective	4692 (73)	298 (49)	6.4 (5.7–7.1)	<0.001
	Emergency	1749 (27)	312 (51)	17.8 (16.1–19.7)	
Number of hospital admissions in previous 5 years	0	2437 (38)	206 (34)	8.4 (7.4–9.6)	0.008
	1	1631 (25)	139 (23)	8.5 (7.2–10.0)	
	2	905 (14)	95 (16)	10.5 (8.6–12.7)	
	3	564 (9)	59 (10)	10.5 (8.1–13.3)	
	4	325 (5)	36 (6)	11.1 (7.9–15.0)	
	≥5	579 (9)	75 (12)	13.0 (10.3–16.0)	
Dukes Stage	A	974 (15)	76 (12)	7.8 (6.2–9.7)	0.062
	B	2284 (35)	209 (34)	9.2 (8.0–10.4)	
	C	2126 (33)	228 (37)	10.7 (9.4–12.1)	
	D	651 (10)	54 (9)	8.3 (6.3–10.7)	
	Not recorded	406 (6)	43 (7)	10.6 (7.8–14.0)	
Cancer Site	Colon	4802 (75)	471 (77)	9.8 (9.0–10.7)	0.113
	Rectum	1639 (25)	139 (23)	8.5 (7.2–10.0)	
Co-morbidities	MI or IHD	510 (8)	69 (11)	13.5 (10.7–16.8)	0.001
	CCF	150 (2)	33 (5)	22.0 (15.7–29.5)	<0.001
	COPD or Asthma	293 (5)	37 (6)	12.6 (9.0–17.0)	0.059
	TIIDM	281 (4)	43 (7)	15.3 (11.3–20.1)	0.001

Values are numbers (%) or median (inter-quartile range)

* test for differences between categories within each variable, with the exception of comorbidities which is compared to patients without any of the stated comorbidities

*SIMD* Scottish Index of Multiple Deprivation, *IHD* ischaemic heart disease, *MI* myocardial infarction, *CCF* congestive cardiac failure, *COPD* chronic obstructive pulmonary disease, *TIIDM* type II diabetes mellitus

The proportion of patients who received post-operative organ support was higher in men compared to women (11 % vs. 8 %, *p* < 0.001), patients aged ≥65 years compared to under 65 (10 % vs. 8 % *p* < 0.001), and in emergency as opposed to elective surgical patients (18 % vs. 6 % *p* < 0.001). Post-operative organ support was provided to 22 % of patients with heart failure and 15 % of patients with type II diabetes compared to 9 % in those who had no major co-morbidity.

**Fig. 2:**
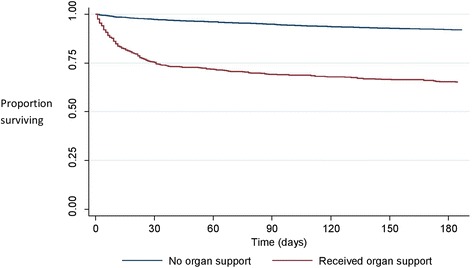
Kaplan Meier survival plot indicating patients who received and did not receive organ support

Hospital mortality for all patients undergoing CRC surgery was 6 %. This was significantly higher in patients who received organ support compared with those who did not (28 % vs. 3 % *p* < 0.001) (Table 2). Patients who received organ support (9 % of all the CRC surgery patients) accounted for 48 % of all in-hospital deaths among CRC surgery patients. Figure 2 shows the Kaplan Meier survival curves for patients who received and did not receive organ support. Six month mortality for all patients was 11 % and was considerably higher in those that received post operative organ support (35 % vs. 8 % *p* < 0.001).

**Table 2:** Hospital and 6 month mortality for patients who did and did not receive organ support

	Hospital Mortality (95 % CI)	6 month mortality (95 % CI)
Received organ support	28 % (24.8–32.1)	35 % (31.0–38.9)
Did not receive organ support	3 % (2.7–3.7)	8 % (7.5–8.9)
*p*-value	<0.001	<0.001

### Factors associated with post operative organ support

Multivariate adjusted odds ratios (OR) for pre-operative factors associated with post operative organ support are shown in Table 3. Age ≥65 (OR 1.37 95 % CI 1.12–1.67), male sex (OR 1.46 95 % 1.22–1.74), and undergoing emergency surgery (OR 3.45 95 % CI 2.88–4.14) were independently associated with receiving organ support following CRC surgery. Additionally patients with CCF (OR 2.07 95 % CI 1.32–3.27) or TIIDM (OR 1.49 95 % CI 1.04–2.14) had an increased likelihood of postoperative organ support on multivariate analysis.

**Table 3:** Predictive factors for receiving organ support following CRC surgery

		Multivariate Analysis	
Baseline Characteristics		Odds Ratio (95 % CI)	*p*-value
Sex	Women	1	
	Men	1.46 (1.22–1.74)	<0.001
Age group at incidence	<65	1	
	≥65	1.37 (1.12–1.67)	0.002
SIMD quintile	1 - most deprived	1	
	2	1.08 (0.85–1.36)	0.53
	3	0.99 (0.77–1.27)	0.92
	4	0.86 (0.65–1.14)	0.29
	5 - least deprived	0.74 (0.55–1.00)	0.05
			0.026*
Number of hospital admissions in previous five years	0	1	
	1	0.98 (0.78–1.24)	0.87
	2	1.18 (0.90–1.55)	0.22
	3	1.10 (0.80–1.52)	0.56
	4	1.18 (0.79–1.77)	0.41
	≥5	1.26 (0.91–1.73)	0.16
Duke’s Stage	A	1	
	B	0.90 (0.68–1.20)	0.47
	C	1.03 (0.78–1.37)	0.83
	D	0.61 (0.42–0.90)	0.012
	Not recorded	0.85 (0.56–1.28)	0.43
Cancer Site	Colon	1	
	Rectum	1.08 (0.88–1.34)	0.46
Nature of Surgery	Elective	1	
	Emergency	3.45 (2.88–4.14)	<0.001
Major Comorbidities	No CHD/MI, CCF, COPD/asthma, TIIDM	1	
	CHD/MI	1.00 (0.72–1.38)	1
	CCF	2.07 (1.32–3.27)	<0.001
	COPD/asthma	1.04 (0.71–1.53)	0.83
	TIIDM	1.49 (1.04–2.14)	0.032

* test for trend

*SIMD* Scottish Index of Multiple Deprivation, *IHD* ischaemic heart disease, *MI* myocardial infarction, *CCF* congestive cardiac failure, *COPD* chronic obstructive pulmonary disease, *TIIDM* type II diabetes mellitus

Increasing SIMD quintile (less social deprivation) (OR 0.93 95 % CI 0.88–0.99) and having metastatic disease (OR 0.61 95 % CI 0.42–0.90) were independently associated with a decreased likelihood of admission to ICU for post-operative organ support.

**Fig. 3:**
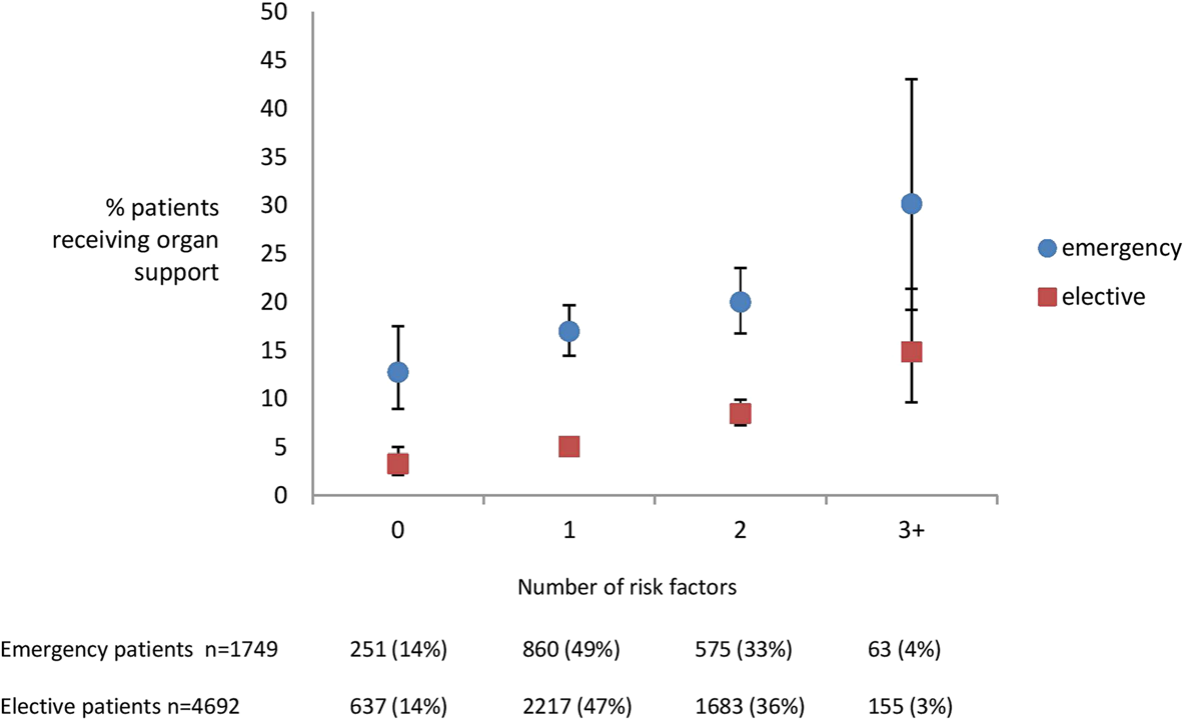
Percent of patients receiving organ support according to number of risk factors

We assessed the effect the total number of risk factors had on the risk of post-operative organ support. With an increasing number of risk factors, there was an increase in the proportion of patients who received post-operative organ support. Only 3 % of those patients with no identified risk factors required post-operative organ support compared with 31 % in those with 4 or more risk factors. In patients with no known risk factors, 3 % of elective patients received post-operative organ support compared with 13 % of those undergoing emergency surgery (Fig. 3). For both elective and emergency surgery patients, an increasing number of risk factors resulted in an increased proportion of patients in receipt of organ support.

### Organ support received

A total of 610 patients received at least one form of organ support following CRC surgery with mechanical ventilation being the commonest. It was required in 8 % of all CRC surgery patients post-operatively; thus 84 % of CRC patients who received any form of organ support, required ventilation. Seven percent of all patients (72 % of organ support patients) received vasoactive drugs and 1 % (11 %) renal replacement therapy.

#### Dukes staging

A greater proportion of emergency patients presented with Dukes D disease compared with elective surgery (18 % vs. 7 %, *p*-value <0.001). On multivariate analysis Dukes stage D patients were less likely to receive organ support than patients with stage A disease OR 0.61 (95 % CI 0.42–0.9, p 0.012). However, once admitted to ICU for organ support Dukes staging did not appear to affect the level of organ support. The proportion of Dukes A and Dukes D patients receiving more than one form of organ support were similar at 57 and 58 % respectively (*p* = 0.596). Although there was no difference in the proportions of patients receiving mechanical ventilation (Dukes A 76 % versus Dukes D 89 %, *p* = 0.069) or renal replacement therapy (Dukes A 16 % versus Dukes D 9 %, *p* = 0.277), only 63 % of patients with Dukes Stage D received vasoactive drugs compared with 80 % of those with Dukes stage A (*p* = 0.028).

#### Elective versus emergency surgery

Of patients receiving organ support, 55 % of elective surgery patients received more than one form of organ support compared with 59 % in the emergency surgery group (*p* = 0.289) yet only 7 % of emergency patients received 3 forms of organ support vs. 13 % of elective surgery patients (*p* = 0.014). Seventy-nine percent of elective patients received mechanical ventilation compared with 89 % of emergency surgery patients (*p* = 0.001). Elective surgery patients were more likely to receive RRT compared with emergency surgery patients at 14 and 8 % respectively (*p* = 0.023), however, there was no difference in the receipt of vasoactive drugs between emergency versus elective patients (76 % vs. 70 % respectively, *p* = 0.119).

## Discussion

Post-operative organ dysfunction significantly alters the surgical course, recovery and mortality from CRC surgery. This study has found that age ≥65, male sex, emergency surgery, increasing social deprivation, congestive cardiac failure and Type II Diabetes Mellitus increase the likelihood of organ support being provided in the perioperative period. We found that nearly 10 % of CRC surgical patients received post-operative organ support with an associated increase in hospital mortality of almost ten-fold (28 % versus 3 % respectively).

In the UK, elective CRC patients have standardised care pathways and optimisation prior to surgery in a way that cannot exist for the emergency patient. They have been assessed and counselled with regards to the level of risk and ‘fitness for theatre’. The finding that emergency patients are more likely to require organ support than elective patients post-operatively (18 % versus 6 %) reflects this disparity. Emergency patients are more likely to present with an acute abdominal insult, which may be preceded by a period of illness. The delayed presentation is apparent in our study by the finding that a greater proportion of emergency patients had Dukes D disease compared with the elective surgical population (18 % vs 7 %, *p*-value <0.001). Advanced cancer presentation often results in patients with poorer physiological reserve and prognosis [25] and may explain why elective patients were more likely to receive 3 forms of organ support compared with emergency surgery (13 % vs 7 %, *p* = 0.014). Thus it is not surprising that prior studies have demonstrated that emergency surgery is a poor prognostic factor both in terms of short and long term survival [33, 34].

It is difficult to attribute pathophysiological mechanisms to each individual risk factor. However male gender being found to increase likelihood of organ support may be explained, at least in part, by surgery in the anatomically narrower male pelvis being more technically challenging thus leading to a potential increase in the likelihood of anastomotic leak.

Metastatic disease was associated with a decreased likelihood of receiving organ support following CRC surgery. It seems doubtful that those patients with advanced disease are less likely to develop organ failure and instead we would postulate that these patients have been considered inappropriate candidates for organ support and ICU intervention. In general there is a reluctance to expose these patients to the aggressive and unpleasant treatment of ICU and instead focus care towards palliation [35].

Further research is required to address factors that influence both the short and longer-termsurvival in CRC patients in ICU in order to provide a comprehensive description of these patients. The findings that certain co-morbidities such as CCF and TIIDM increase the likelihood of receiving organ support following CRC surgery allow us to identify at risk individuals. There may also be a role for optimising the management of these conditions prior to and following surgery to improve outcomes in this group of patients. In addition it might allow the opportunity for discussions regarding advanced directives to take place.

### Strengths and limitations

The assessment of the effect of nature of surgery is a potential drawback of this study. Due to the nature of the database a number of patients who received an “emergency surgery” may have initially been admitted for an elective surgery in the same hospital admission. Nevertheless, this group of patients is still representative in showing the effect that unplanned surgery has on perioperative outcomes even if as a patient group they differ slightly from the literature’s traditional description of emergency CRC surgery patients.

Although analysed retrospectively, this study has been performed using prospectively collected data that is entered into national databases. Within the West of Scotland, every ICU patient is admitted onto the Wardwatcher database and every new cancer diagnosis is entered into the Cancer Registry thus ensuring we capture the appropriate population. The unique identifier (CHI) used in Scotland also makes data linkage more robust. The main limitation of using databases is the level of detail contained within them and the accuracy of the data entry. However, we believe that this study of over 6000 cancer patients highlights the significant morbidity and mortality facing nearly 10 % of CRC patients in the perioperative period. This may allow for better risk assessment and optimisation in the perioperative period.

## Conclusions

Nearly one in ten patients undergoing CRC surgery receive organ support in the post operative period. This was associated with a significant increase in mortality with organ support patients having a hospital mortality close to ten times that of those without organ support. We identified several risk factors which increase the likelihood of receiving organ support post operatively including advanced age, emergency surgery, male sex and certain co-morbidities. These appear to have an additive effect with an increased likelihood of organ support being received with each additional risk factor. Emergency surgery had the greatest impact in terms of impact on requirement for post-operative organ support. Post operative organ support is not routinely discussed with patients undergoing CRC surgery, however, given the frequency that this occurs and the significant impact upon outcomes we suggest that this should considered in patients with features associated with increased risk.

## Abbreviations

CRC: Colorectal cancer
ICU: Intensive care unit
ASA: American Society of Anaesthesiologists
ICD: International classification of disease
ISD: Information services division
SMR: Standardised mortality ratio
SICSAG: Scottish Intensive Care Society Audit Group
RRT: Renal replacement therapy
SIMD: Scottish index of multiple deprivation
MI: Myocardial infarction
CHD: Coronary heart disease
CCF: Congestive cardiac failure congestive cardiac failure
COPD: Chronic obstructive pulmonary disease
TIIDM: Type II diabetes mellitus
OR: Odds ratio
